# Factors influencing bioequivalence evaluation of insulin biosimilars based on a structural equation model

**DOI:** 10.3389/fphar.2023.1143928

**Published:** 2023-04-03

**Authors:** Huarui Shao, Yi Tao, Chengyong Tang

**Affiliations:** ^1^ College of Pharmacy, Chongqing Medical University, Chongqing, China; ^2^ Phase I Clinical Research Center, The First Affiliated Hospital of Chongqing Medical University, Chongqing, China; ^3^ Phase I Clinical Research Center, Bishan Hospital of Chongqing Medical University, Chongqing, China

**Keywords:** insulin biosimilars, bioequivalence, glucose clamp technique, drug clinical trials, structural equation model

## Abstract

**Objective:** This study aimed to explore the factors affecting the bioequivalence of test and reference insulin preparations so as to provide a scientific basis for the consistency evaluation of the quality and efficacy of insulin biosimilars.

**Methods:** A randomized, open, two-sequence, single-dose, crossover design was used in this study. Subjects were randomly divided into TR or RT groups in equal proportion. The glucose infusion rate and blood glucose were measured by a 24-h glucose clamp test to evaluate the pharmacodynamic parameters of the preparation. The plasma insulin concentration was determined by liquid chromatography–mass spectrometry (LC-MS/MS) to evaluate pharmacokinetic parameters. WinNonlin 8.1 and SPSS 23.0 were applied for PK/PD parameter calculation and statistical analysis. The structural equation model (SEM) was constructed to analyze the influencing factors of bioequivalence by using Amos 24.0.

**Results:** A total of 177 healthy male subjects aged 18–45 years were analyzed. Subjects were assigned to the equivalent group (N = 55) and the non-equivalent group (N = 122) by bioequivalence results, according to the EMA guideline. Univariate analysis showed statistical differences in albumin, creatinine, T_max_, bioactive substance content, and adverse events between the two groups. In the structural equation model, adverse events (*β* = 0.342; *p* < 0.001) and bioactive substance content (*β* = −0.189; *p* = 0.007) had significant impacts on the bioequivalence of two preparations, and the bioactive substance content significantly affected adverse events (*β* = 0.200; *p* = 0.007).

**Conclusion:** A multivariate statistical model was used to explore the influencing factors for the bioequivalence of two preparations. According to the result of the structural equation model, we proposed that adverse events and bioactive substance content should be optimized for consistency evaluation of the quality and efficacy of insulin biosimilars. Furthermore, bioequivalence trials of insulin biosimilars should strictly obey inclusion and exclusion criteria to ensure the consistency of subjects and avoid confounding factors affecting the equivalence evaluation.

## 1 Introduction

With the development of biotechnology, biologics have shown irreplaceable effects in therapeutic areas (Zinzani et al., 2019; Akram et al., 2021). With the expiration of a large number of patent protections for original biologics, biosimilars have ushered in important development opportunities (Nabhan et al., 2018; Thill et al., 2019; Akram et al., 2021) as generic drugs of biological drugs, which are therapeutic biological products that are similar to reference drugs in terms of quality, safety, and efficacy (Curigliano et al., 2016). In December 2006, the European Medicines Agency (EMA) approved the world’s first biosimilar recombinant human growth hormone (somatropin, Sandoz) and successively issued guidelines for biosimilars in various fields (Calvo et al., 2018). In February 2019, the National Medical Products Administration (NMPA) approved China’s first rituximab injection (Henlius) (Zi-yue, 2021). At present, biosimilars have achieved great progress in the research and development field around the world. Because of the relatively low price and high accessibility of biosimilars, they can better satisfy people’s needs (de Mora, 2019).

Insulin and its biosimilars are indispensable for patients with diabetes (Vencio et al., 2022). As of 2020, China accounts for the largest number of people with diabetes (114 million), which is the largest proportion in the world (Luo et al., 2020). Timely initiation of exogenous insulin supplementation therapy is a necessary hypoglycemic management strategy (American Diabetes Association, 2021). Insulin biosimilars can better simulate the human physiological state of insulin secretion patterns, reduce the risk of hypoglycemia, achieve a more flexible dosing time, improve patient compliance, and reduce diabetic complications (Hilgenfeld et al., 2014). Due to the fact that long-acting insulin has a stable hypoglycemic effect, small inter-individual and intra-individual differences, high reproducibility in the daytime, and a low risk of nocturnal hypoglycemia, researchers are committed to the research and development of new long-acting insulin biosimilars (Tibaldi, 2014). In 1996, the FDA approved the listing of the first recombinant human insulin analog, insulin lispro (Humalog) (Eli Lilly, United States) (Owens et al., 2022). Subsequently, various insulin biosimilars have been approved for marketing, providing more treatment options for hypoglycemic management. Insulin glargine and insulin degludec, both being long-acting and safe, can stably control blood glucose. Insulin lispro 25R and insulin lispro 50R are premixed insulins, taking into account the needs of patients for basic and meal insulin. These insulins are widely used in the treatment of diabetes with their huge clinical advantages and win a large market share.

The listing of biosimilar drugs usually needs to implement pharmacokinetic and pharmacodynamic evaluations (FDA, 2016). A bioequivalence study was recommended by national regulatory agencies at home and abroad to verify the consistency of two preparations (FDA, 2015; NMPA, 2015; PMDA, 2015; EMA. Guideline, 2018). The geometric means of the 90% CI of the main PK/PD evaluation indicators of test and reference preparations were within the accepted range of 80.00%–125.00% and were regarded as bioequivalent (FDA, 2016; NMPA, 2022). Inhibition of endogenous insulin was a key point in the equivalent evaluation of insulin biosimilars; the euglycemic clamp technique was considered the best available method for measuring the action of insulin, and the degree of serum C-peptide inhibition was the most appropriate and accurate marker for assessing endogenous insulin suppression levels (Tao et al., 2021). In addition to verifying similar physicochemical properties and functional characteristics, similar pharmacokinetic (PK) and pharmacodynamic (PD) *versus* time profiles were considered the most prominent proof of similar absorption, metabolism, and efficacy of two preparations (EMA, 2013). The EMA guidelines recommend that the main PK evaluation index for long-acting insulin biosimilars is AUC_0-τ_, and the main PD evaluation index is AUC_GIR0-τ_ (EMA, 2013).

Bioequivalence evaluation is the established way for the review of generic drugs and occupies an important position in generic drug applications (Andrade, 2015). At the same time, it was also inevitable in the development of new drugs in production processes and dosage form changes (Gámez-Belmonte et al., 2018). Biosimilars and reference formulations must be bioequivalent in safety and efficacy (Schellekens, 2002). Although national regulatory agencies have paid more attention to the research and development of biosimilars and equivalence research, drawbacks still exist (Wolff-Holz et al., 2019). Due to the complexity of biosimilars and the diversity of influencing factors for bioequivalence test results, the clinical trial evaluation system and equivalence determination criteria for biosimilars should be continuously improved.

There are many factors affecting insulin preparations’ bioequivalence evaluation, including the physicochemical properties of the drug itself, the effect of the preparation, and subjects’ physiological status (Karalis et al., 2008). For insulin injections, a certain factor affecting the absorption rate is the subcutaneous blood flow (SBF) at the injection site (Søeborg et al., 2009). SBF is influenced by a complex interaction of factors such as the injection site, body temperature, exercise, obesity, postural position, blood pressure, vasodilation/vasoconstrictor drug use, and smoking. An increase in SBF accelerates insulin absorption, which can be increased by body temperature and exercise. Increased skin temperature and exercise can accelerate insulin absorption. Inversely, obesity and smoking can decrease insulin absorption, fat hypertrophy or obesity leads to reducing insulin absorption, and smoking can cause peripheral vasoconstriction and delays insulin absorption (Gradel et al., 2018). Studies have found that blood glucose levels also have an effect on insulin absorption (Fernqvist-Forbes et al., 1988). Many of these factors have also been reported to influence the pharmacokinetic profile of insulin (Cengiz et al., 2014). In addition, because of inter-individual differences in the distribution, degradation, and clearance of insulin, factors such as age, sex, and weight may affect insulin pharmacokinetics (Fernqvist-Forbes et al., 1988). When evaluating the bioequivalence of insulin and its biosimilars, the variations of individuals and influences of these factors could not be ignored.

The structural equation model (SEM) is an important statistical tool for multivariate analysis, which is a statistical method to analyze the relationship between variables based on the covariance matrix of variables (Rappaport et al., 2020). Compared with general regression analysis, the structural equation model can control the measurement error better and ensure higher parameter estimation accuracy. It also supports the construction of complex multivariable models, which can simultaneously estimate the factor structure and factor relationship, and have abundant fitting evaluation indexes to evaluate the models (Fu, 2020; Fang et al., 2022; Wei, 2022).

In this study, the main factors associated with the consistency evaluation of the quality and efficacy of insulin and its biosimilars were considered. The demographic characteristics, vital signs, biochemical indicators, PK indicators, PD indicators, safety indicators, and preparation factors were included to construct the consistency evaluation model. We aimed to explore the factors affecting the bioequivalence of two preparations using a multiple statistical model, thus providing a scientific basis for the review and approval of insulin biosimilars.

## 2 Materials and methods

### 2.1 Study design

This study comes from four insulin bioequivalence trials, including insulin glargine injection (CTR20191031), insulin degludec injection (CTR20201129), insulin lispro injection 25R (CTR20211981), and insulin lispro injection 50R (CTR20211612); all studies were approved by the Ethics Committee of the First Affiliated Hospital of Chongqing Medical University (20190101, 20198402, 20219901, and 20215602). A randomized, open, two-sequence, single-dose, crossover design was used in this study. Subjects were randomly divided into TR or RT sequence (the test preparation was injected in the first cycle and the reference preparation in the second cycle, or *vice versa*) groups in equal proportion. Subjects were provided with a uniform standard dinner on the day before each cycle and fasted overnight. Fasting was followed for at least 10 h before administration and until 24 h after administration (fasting without water). The dosages of insulin glargine and insulin degludec were both 0.4 U/kg, and the dosages of insulin lispro 25R and insulin lispro 50R were 0.3 U/kg. The washout period during two cycles was 7–14 days.

The glucose infusion rate and blood glucose were measured by a 24-h glucose clamp test to evaluate the pharmacodynamic parameters of the preparation. Subjects were given intravenous access to both arms before administration. One side was used for blood collection, and the other side was given a 20% glucose solution. A measure of 2 mL of venous blood was collected at each blood collection point to measure serum C-peptide concentrations to evaluate endogenous insulin inhibition and the quality of clamp study, and 4 mL of venous blood was collected at each point for the detection of the plasma insulin concentration to evaluate PK parameters. Venous blood was collected not exceeding 0.5 mL at each point to measure the blood glucose to evaluate PD. The C-peptide and PK sampling points of insulin glargine were 20 min before administration, 0 min, 0.5, 1, 2, 3, 4, 5, 6, 8, 10, 12, 15, 18, 21 and 24 h; PD sampling points were 30, 20, and 10 min before administration; and 0 min, every 10 min from 0 to 8 h, every 20 min from 8 to 15 h, and every 30 min from 15 to 24 h. The C-peptide and PK sampling points of insulin degludec were 0.5 h before administration, 0.25, 1, 2, 4, 5, 6, 8, 10, 12, 14, 24, 36, 48, 72, and 96 h; PD sampling points were 30, 20, and 10 min before administration; and 0 min, every 10 min from 0 to 12 h, every 20 min from 12 to 18 h, and every 30 min from 18 to 24 h. The PK sampling points of insulin lispro 25R and insulin lispro 50R both were 30 min before administration, 0 min, 10, 20, 30, 40, 50, 60, 70, 80, 90, 100, 110, 120, 150, 180, 210, 240, 300, 360, 420, 480, 600, 720, 840, 960, 1,200, and 1,440 min; and the PD sampling points both were 30, 20, and 10 min before administration; and 0 min, every 5 min from 0 to 2 h, every 10 min from 2 to 8 h, every 20 min from 8 to 16 h, and every 30 min from 16 to 24 h. C-peptide sampling points of insulin lispro 25R were 30 min before administration, 0 min, 60, 120, 240, 360, 480, 600, 720, 840, 960, 1200, and 1440 min. C-peptide sampling points of insulin lispro 50R were 30 min before administration, 0 min, 10, 20, 30, 40, 50, 60, 90, 120, 180, 240, 300, 360, 420, 480, 600, 720, 840, 960, 1200, and 1440 min. The mean values of C-peptide and blood concentrations 20 min/30 min and 0 min before administration were the baseline values of insulin and C-peptide, respectively. The target blood glucose values of subjects were determined by subtracting 0.28 mmol·L-1 from their baseline average blood glucose values of 30, 20, and 10 min before administration.

Blood samples were placed in a cryogenic centrifuge within 60 min, centrifuged at 2000 *g* at 2°C–8°C for 10 min, and the plasma and serum were separated and stored in a refrigerator at 60 °C. Serum C-peptide levels were measured by an enzyme-linked immunosorbent assay (ELISA). The plasma insulin concentration was determined by liquid chromatography–mass spectrometry (LC-MS/MS), and the lower limit of quantitation was 0.07 ng/mL. The plasma concentration of insulin glargine was determined as the sum of the concentrations of the prototype insulin glargine drug, insulin glargine metabolite M1 (21A-glycine-insulin), and insulin glargine metabolite M2 (21A-glycine de-30B-threonine-insulin). Plasma concentrations of insulin degludec, insulin lispro 25, and insulin lispro 50R were detected as prototype insulin drugs. Blood glucose concentration was immediately analyzed using an automatic glucose oxidase analyzer Biosen C-line GP+(Germany) during clamping. Based on the blood glucose test results, the infusion rate of a 20% glucose solution was adjusted to maintain the subject’s blood glucose level within ±10% of the target blood glucose value, and the glucose infusion rate (GIR) was calculated.

The safety evaluation was based on the Common Terminology Criteria for Adverse Events (NCI-CTCAE5.0). All subjects were observed to have any adverse events during the clinical study, including clinically significant abnormalities in clinical symptoms, vital signs, laboratory tests, hypoglycemic reactions, and injection site reactions. The clinical manifestations, severity, relevance to the drug, occurrence time, end time, duration, treatment measures, and outcomes were recorded.

To be eligible, subjects needed to: 1) be aged 18 to 45; 2) have a BMI of 19–24 kg/m2; 3) be without diabetes, insulin resistance, and family history of diabetes; 4) be without cardiovascular disease; 5) be non-smokers and non-alcohol abusers: 6) be free of abnormalities in blood and urine routine examinations, hepatic and renal function tests, or electrocardiograms; 7) complete two-period trials. All volunteers have written informed consent previously, and enrolled subjects were applied to the inclusion and exclusion criteria of study protocols. We carried out the trial in accordance with the principles of the Declaration of Helsinki. According to the EMA guideline, there are four bioequivalence evaluation indicators, and six main indicators that may affect bioequivalence evaluation were screened. Incomplete data were excluded through analysis.

### 2.2 Structural equation model

There are two kinds of variables in a structural equation model: one is an explicit variable, which can be measured directly and is the observation index in the model; the other is latent variables, which are not directly observed variables and are reflected by their corresponding explicit variables. Structural equation models are divided into measurement models (the relationship between explicit variables and latent variables) and structural models (the relationship between latent variables). This study analyzed the influence of each latent variable on the evaluation of bioequivalence. The SEM model fit criteria used were root mean square error of approximation (RMSEA) < 0.08, comparative fit index (CFI) > 0.90, and Tucker–Lewis index (TLI) > 0.9.

### 2.3 Statistical analysis

WinNonlin 8.1 and SPSS 23.0 were applied for PK/PD parameter calculation and statistical analysis. Parameter estimates were computed by non-compartmental analysis (NCA) of the total insulin concentration *versus* time profiles and glucose infusion rate *versus* time profiles, and the pharmacokinetic parameters included the area under the plasma concentration curve from administration to end of clamp at time 24 h (AUC_0–24h_), area under the plasma concentration curve extrapolated to infinite time (AUC_0–∞_), maximum plasma concentration (C_max_), plasma concentration half-life (T_1/2_), and time to Cmax (T_max_). The pharmacodynamic parameters were the area under the glucose infusion rate curve from administration to end of clamp at time 24h (AUC_GIR0–24h_), area under the glucose infusion rate curve extrapolated to infinite time (AUC_GIR0–∞_), peak of glucose infusion rate (GIRmax), and time to GIRmax (tGIRmax). According to the current bioequivalence evaluation standard and the guidelines (EMA, 2013; NMPA, 2022), AUC_0–24h_, C_max_, AUC_GIR0–24h,_ and GIR_max_ were adopted to evaluate bioequivalence. Here, 90% CI of the geometric mean ratio of the parameters in the range of 80.00%–125.00% was regarded as bioequivalent; participants were divided into equivalent group and non-equivalent group by bioequivalence results. All variables were described using descriptive statistics. For continuous variables conforming to a normal distribution, the values were expressed as the mean ± standard deviation (SD); however, in a skew distribution, the values were expressed as the median (inter-quartile range). Some data were naturally log-transformed prior to analysis. Groups were compared using two independent sample t-tests and the Wilcoxon rank-sum test. The SEM was adopted to analyze the influencing factors of bioequivalence; the SEM was performed through Amos 24.0. All the tests were performed using a two-sided test with a *p* < 0.05 as the statistical difference.

## 3 Results

**FIGURE 1:**
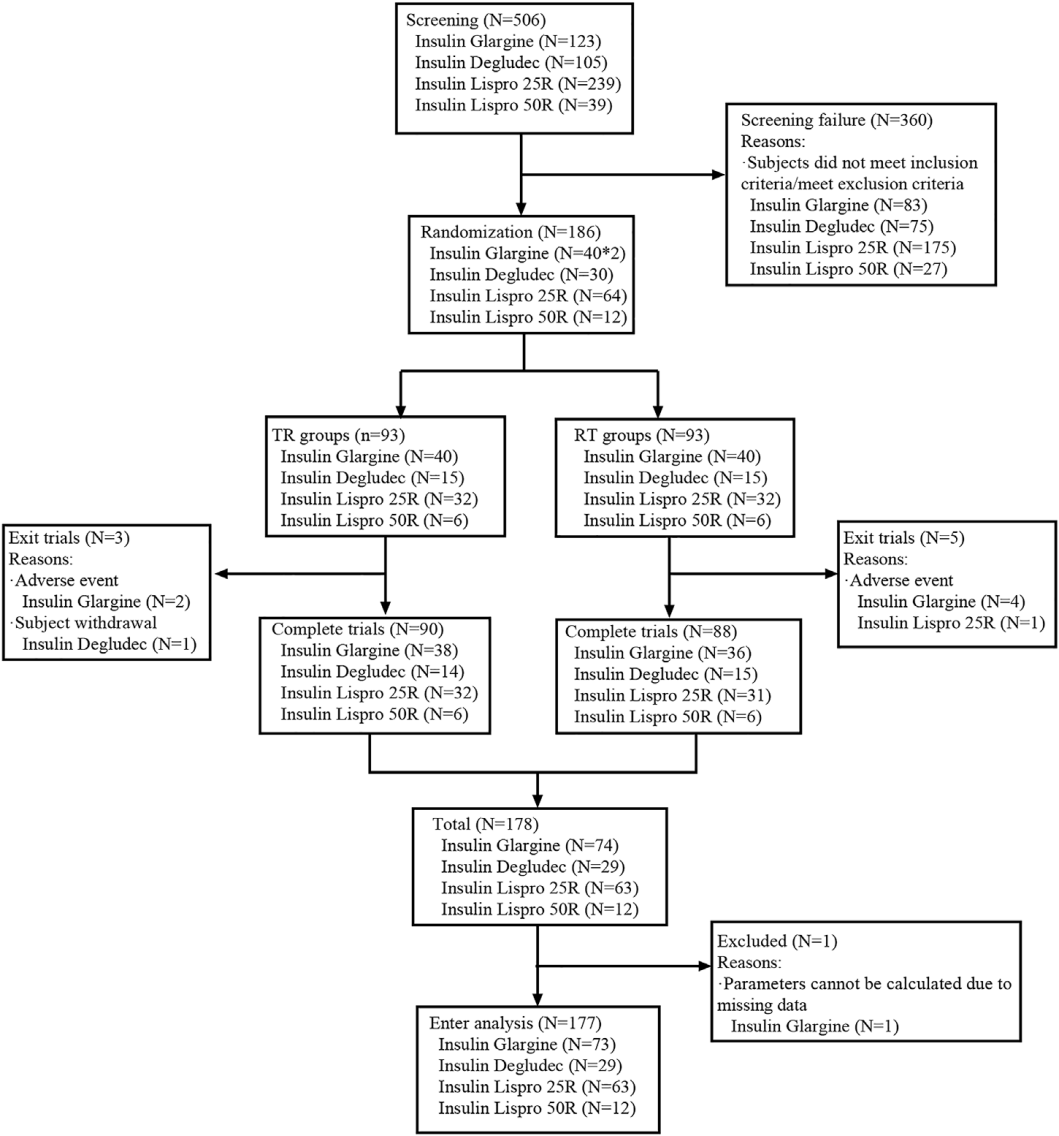
Flowchart of subjects enrolled in this study (insulin glargine carried out a four-cycle cross-design trial with two sets of data).

A total of 506 subjects were screened, and 177 of them were analyzed in the study (Figure 1).

### 3.1 Quality of the clamp study

**FIGURE 2:**
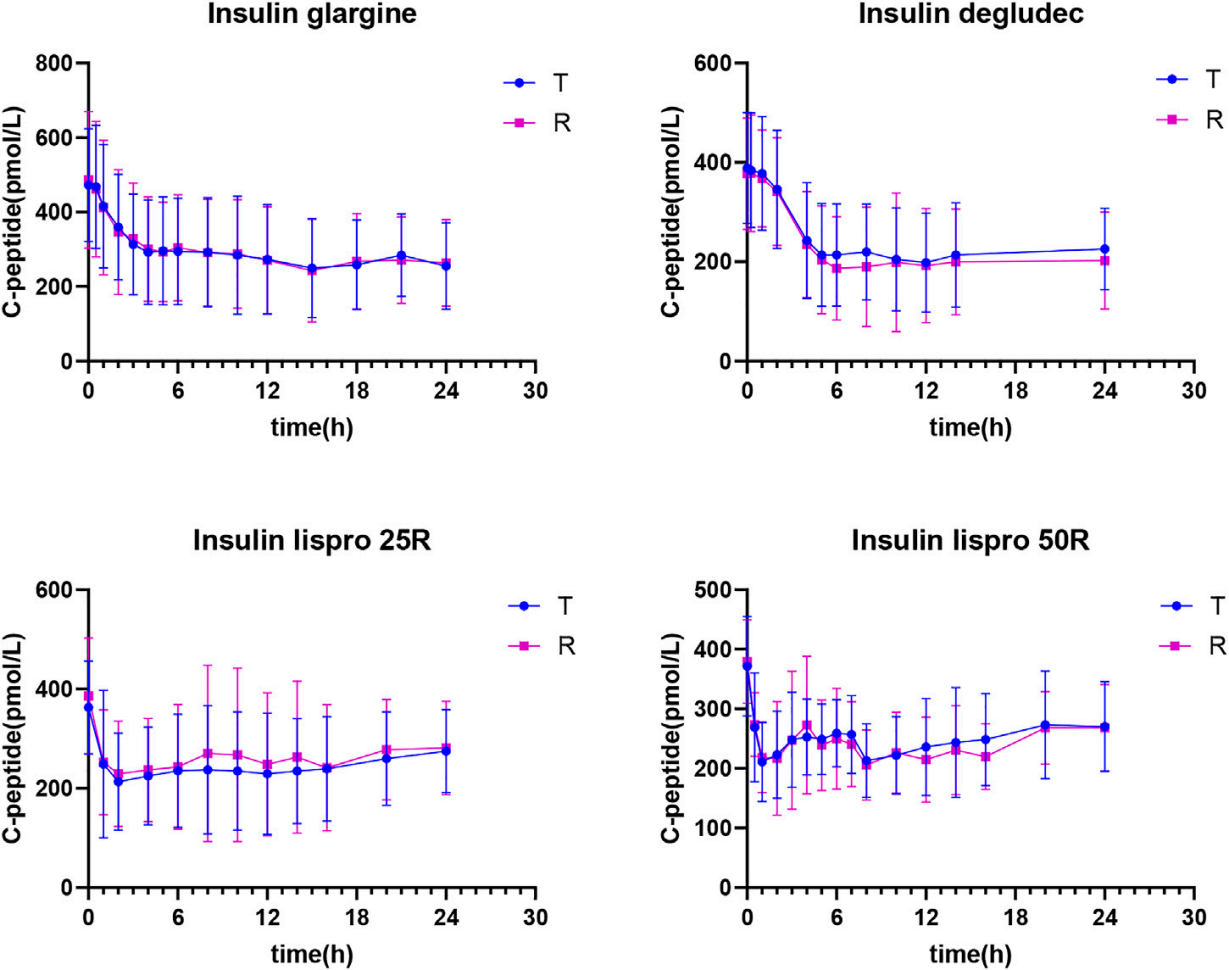
Mean C-peptide concentration *versus* time profiles after insulin biosimilar administration in healthy volunteers.

Endogenous insulin secretion was restrained by euglycemic clamps, and the serum C-peptide levels were used to reflect the degree of restriction. The profiles of C-peptide changes over time are shown in Figure 2, where C-peptide showed a descending trend in all test and reference insulin preparations and the endogenous insulin secretion in these four insulin preparations was well-restrained during the clamp. The clamp studies were of superior quality.

### 3.2 PK curve

**FIGURE 3:**
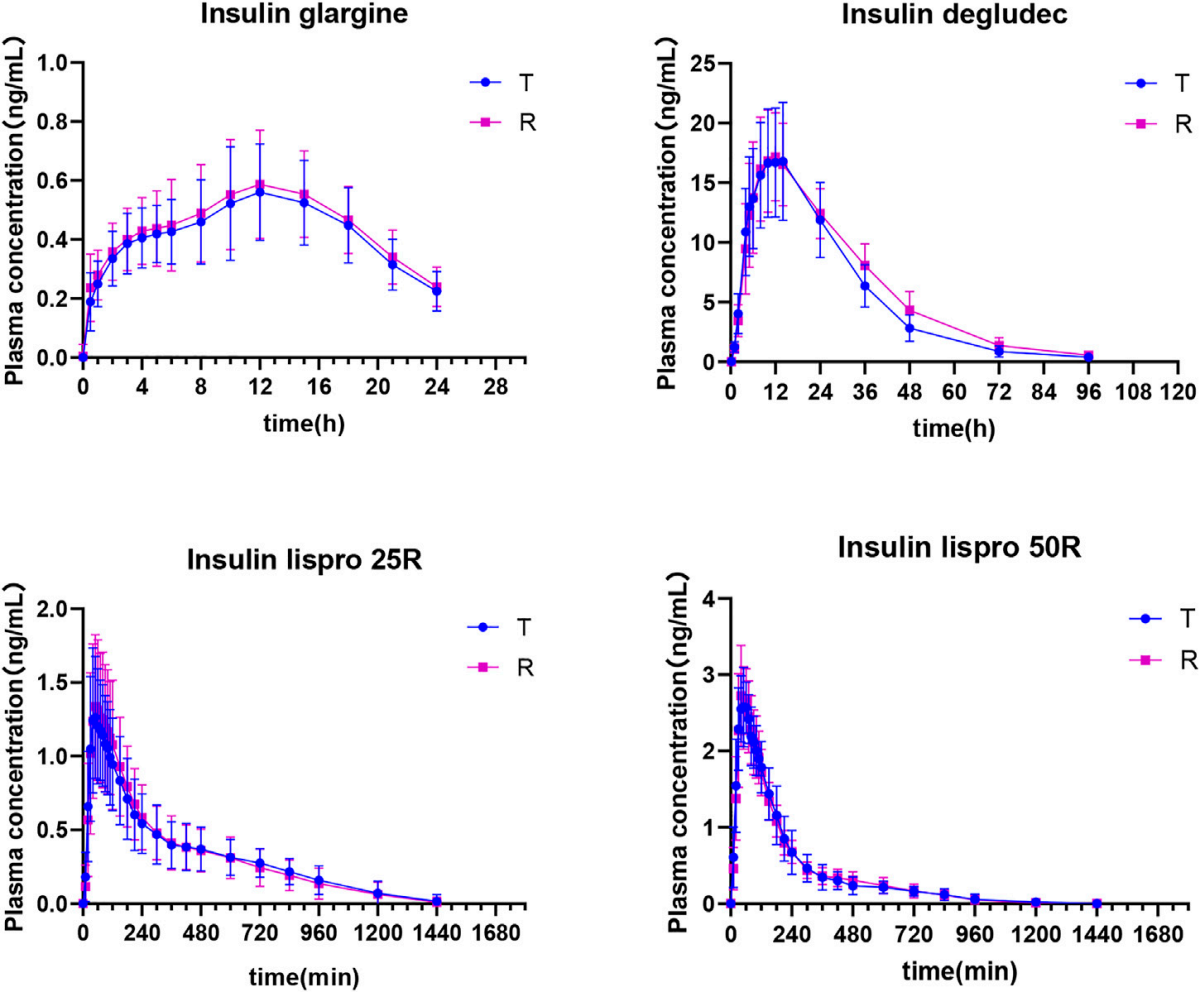
Plasma concentration versus time profiles after insulin biosimilar administration in healthy volunteers.

The changes in plasma insulin concentration overtime are shown in Figure 3. The PK curves of all insulin biosimilars fitted well and presented obvious peaks. C_max_ of each biosimilar could be measured.

### 3.3 Univariate analysis of factors affecting bioequivalence

**FIGURE 4:**
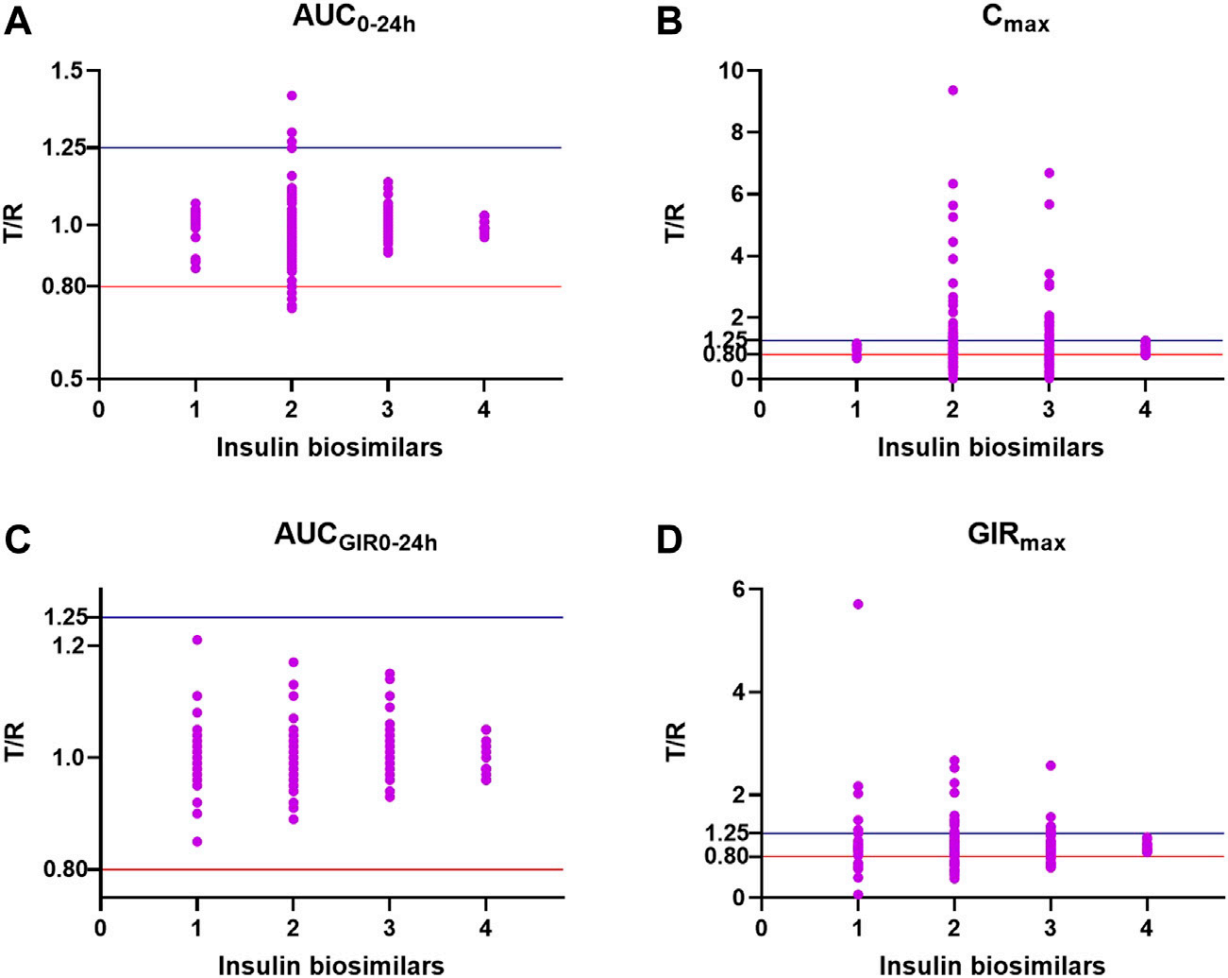
Bioequivalence evaluated in AUC_0–24h_ **(A)**, C_max_ **(B)**, AUC_GIR0–24h_ **(C),** and GIR_max_ **(D)**. The *y*-axis represents T/R values, and the *x*-axis represents insulin biosimilars (1. insulin glargine injection; 2. insulin degludec injection; 3. insulin lispro injection 25R; 4. insulin lispro injection 50R).

A final total of 177 participants were analyzed in this study, of whom 55 individuals (31.07%) had a T/R ratio completely inside the bioequivalent interval (80%–125%). The bioequivalence evaluation of four insulins in each of the PK/PD parameters is shown in Figure 4; Except AUC_GIR0-24_, the other three parameters AUC_0–24 h_, C_max_, and GIR_max_ have individual inequivalence. Demographic characteristics did not differ between the two groups, and the mean age of the subjects was 27.41 ± 5.04. The differences of influencing factors on bioequivalence are shown in Table 1. The results showed statistically significant differences in albumin, creatinine, T_max_, bioactive substance content, and adverse events.

**TABLE 1:** Univariate analysis of bioequivalence and influencing factors.

Variable	Equivalent group (*n* = 55)	Non-equivalent group (*n* = 122)	Total (*n* = 177)	*p*
Demographic characteristics				
Age	28.02 ± 5.47	27.13 ± 4.82	27.41 ± 5.04	0.279
BMI	21.79 ± 1.47	22.03 ± 1.31	21.96 ± 1.36	0.284
Nation				0.603
Han	51 (92.73%)	117 (95.90%)	168 (94.92%)	
Others	4 (7.27%)	5 (4.10%)	9 (5.08%)	
Vital signs				
SBP	120.76 ± 9.14	119.75 ± 9.10	120.07 ± 9.10	0.496
DBP	74.35 ± 7.53	74.48 ± 7.66	74.44 ± 7.60	0.917
HR	73.82 ± 10.30	76.43 ± 11.31	75.62 ± 11.04	0.146
T	36.25 ± 0.43	36.17 ± 1.90	36.31 ± 0.46	0.750
Biochemical parameters				
AST	18.15 ± 4.86	17.59 ± 5.18	17.76 ± 5.07	0.502
ALT	18.02 ± 8.55	19.98 ± 9.31	18.68 ± 9.06	0.513
TP	72.75 ± 3.75	74.07 ± 4.63	73.66 ± 4.41	0.063
ALB	48.78 ± 2.46	49.92 ± 2.09	49.57 ± 2.27	0.002
SCR	78.64 ± 9.16	81.37 ± 7.90	80.52 ± 8.38	0.044
Glu	4.97 ± 0.41	5.05 ± 0.34	5.03 ± 0.36	0.150
Pharmacokinetic parameters				
AUC_0-∞_*	0.99 (0.97–1.02)	0.99 (0.94–1.03)	0.99 (0.96–1.03)	0.443
T_1/2_*	0.93 (0.71–1.44)	1.02 (0.73–1.42)	0.96 (0.72–1.43)	0.309
T_max_*	1.11 (0.86–1.33)	1.00 (0.80–1.18)	1.00 (0.80–1.22)	0.020
Pharmacodynamics parameters				
AUC_GIR0-∞_*	1.00 (0.98–1.04)	0.99 (0.97–1.03)	1.00 (0.97–1.03)	0.290
tGIR_max_*	1.00 (0.77–1.36)	0.93 (0.65–1.25)	0.96 (0.70–1.27)	0.221
Preparation factors				
Drug content (%)*	98.91 (98.58–102.30)	98.91 (98.58–98.91)	98.91 (98.58–98.91)	0.718
Bioactive substance content (%)*	109.80 (104.40–109.80)	109.80 (104.40–112.44)	109.80 (104.40–112.44)	0.014
Zinc (%)*	95.65 (93.50–103.33)	95.65 (95.65–103.33)	95.65 (95.65–103.33)	0.697
Safety data				
Adverse events (%)*	100.00 (94.00–200.00)	94.87 (94.00–94.87)	94.87 (94.00–94.87)	0.000
Blood routine abnormities	5 (9.09%)	2 (1.64%)	7 (3.95%)	
Blood biochemistry abnormalities	10 (18.18%)	38 (31.15%)	48 (27.12%)	
Urine routine abnormalities	35 (63.64%)	67 (54.92%)	102 (57.63%)	
Injection site reaction	2 (3.64%)	5 (4.10%)	7 (3.95%)	
Anemia	9 (16.36%)	12 (9.84%)	21 (11.86%)	
ECG abnormality	0	11 (9.02%)	11 (6.21%)	
Hypotension	0	1 (0.82%)	1 (0.56%)	
Abdominal pain	1 (1.82%)	0	1 (0.56%)	

Age (year), BMI (Body Mass Index, kg/m^2^), SBP (systolic blood pressure, mmHg), DBP (diastolic blood pressure, mmHg), HR (heart rate, bpm), T (body temperature, °C), AST (aspartate aminotransferase, U/L), ALT (alanine aminotransferase, U/L), TP (total protein, g/L), ALB (albumin, g/L), SCR (creatinine, μmol/L), Glu (glucose, mmol/L). * represents the data were calculated by T/R.

### 3.4 Structural equation model for factors influencing bioequivalence

**FIGURE 5:**
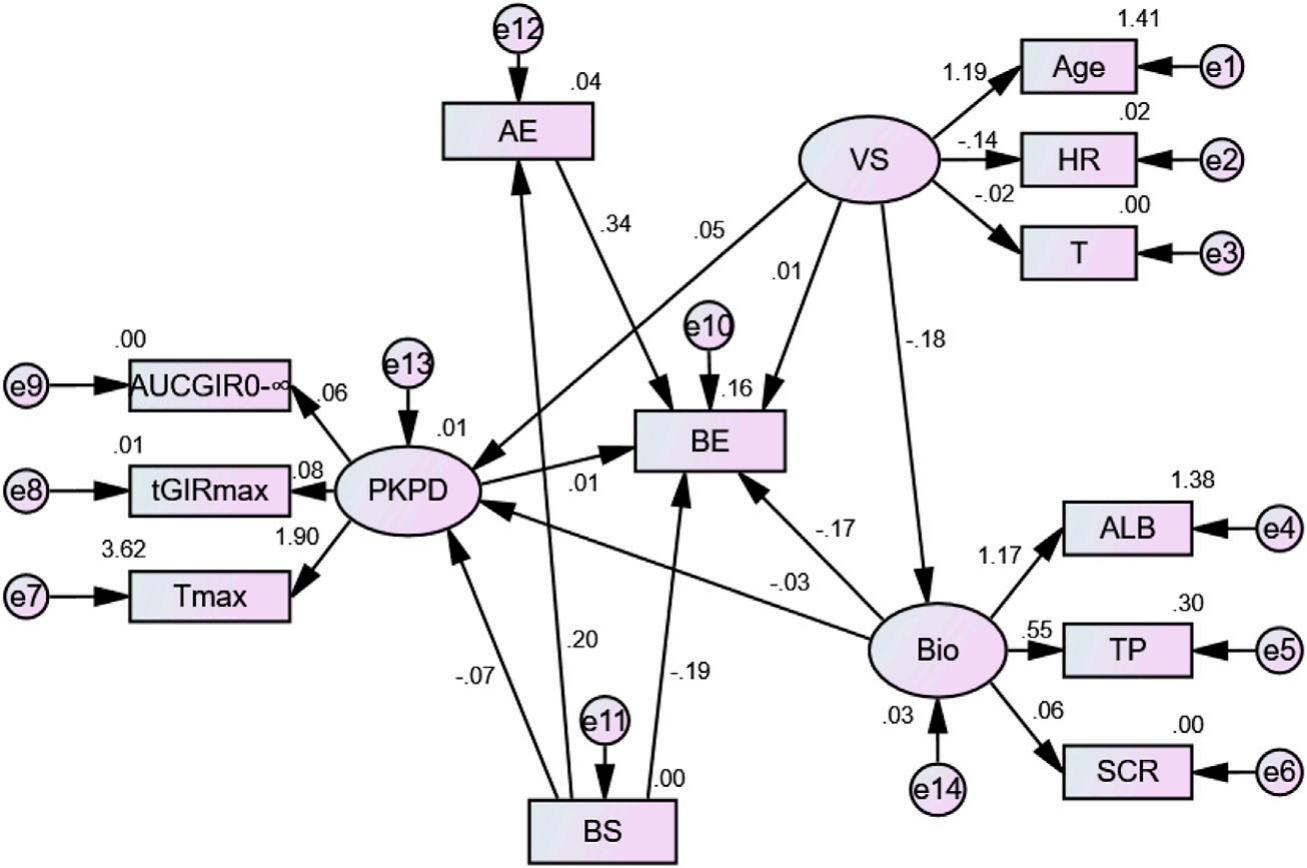
Structural equation model for factors influencing bioequivalence. BE, bioequivalence of two preparations; VS, vital signs; AE, adverse event; PKPD, pharmacokinetic and pharmacodynamic parameters; BS, bioactive substance content; Bio, biochemical parameters; 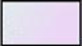  represents the explicit variable; 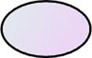  represents the latent variable; and “e” represents the errors.

The model exhibited 6 factors and 10 items (Figure 5), as follows: 1) demographic characteristics and vital signs: age, heart rate, and body temperature; 2) biochemical parameters: albumin, total protein, creatinine; 3) pharmacokinetic and pharmacodynamic parameters: AUC_GIR0-∞_, T_max_, and tGIR_max_; 4) safety data: incidence of adverse events; 5) preparation factors: bioactive substance content; 6) bioequivalence (BE, determined by AUC_0–24h_, C_max_, AUC_GIR0–24h_, and GIR_max_). The model evaluation results showed that the model had a good fit and satisfied the recommended standard requirements (RMSEA = 0.029, CFI = 0.956, and TLI = 0.938).

As seen from Table 2, the structural equation model showed that adverse events (*β* = 0.342; *p* < 0.001) and bioactive substance content (*β* = −0.189; *p* = 0.007) had significant impacts on the bioequivalence of two preparations, and the bioactive substance content significantly affected adverse events (*β* = 0.200; *p* = 0.007). Pharmacokinetic and pharmacodynamic parameters (*β* = 0.012; *p* = 0.899), vital signs (*β* = 0.006; *p* = 0.926), and biochemical parameters (*β* = −0.175; *p* = 0.061) had no significant influence on bioequivalence.

**TABLE 2:** Parameter estimation of the structural equation model.

Influencing factors	Unstandardized estimates	Standard error	Critical ratio	Standardized estimates	*p*-value
AE→BE	0.405	0.083	4.889	0.342	<0.001
BS→BE	−2.256	0.837	−3.624	−0.189	0.007
BS→AE	2.010	0.743	2.706	0.200	0.007
PKPD→BE	0.005	0.043	0.127	0.012	0.899
VS→BE	−0.001	0.005	0.093	0.006	0.926
Bio→BE	−0.030	0.016	−1.874	−0.175	0.061

## 4 Discussion

Biological drugs are mostly produced by living organisms or biologicals, their relative molecular mass is large, and the drug structure and production process are complex, which is difficult to be imitated accurately (Gámez-Belmonte et al., 2018). In addition, in all aspects of the production and circulation of biosimilars, small differences may have greater impacts on the quality, purity, biological properties, and clinical effects of the drug product. This study was the first time to analyze the factors influencing the bioequivalence of insulin biosimilars based on structural equation models and explore the relationships among these factors.

The guidelines for EMA and NMPA recommend the use of AUC_0-τ_ and AUC_GIR0-τ_ as the main evaluation indexes of PK and PD for long-acting insulin biosimilars. In consideration of the stable blood concentration and slow effect of long-acting insulin, C_max_ and GIR_max_ may be difficult to measure and may not be clinically significant (EMA, 2013). In this study, the PK and PD curves of all insulin preparations showed a certain peak; therefore, we also took C_max_ and GIR_max_ as primary indicators to evaluate the speed and extent of drug absorption and action. In addition to the main evaluation indicators, PK indexes such as T_max_ and T_1/2_ reflect the absorption, distribution, metabolism, and excretion of insulin biosimilars in the body. PD indexes such as tGIR_max_ represent the intensity and duration of action *in vivo*. Insulin is a demic endogenous substance, and endogenous islet secretion in healthy volunteers may interfere with exogenous insulin PK and PD measurements, leading to interference in bioequivalence evaluation. It was necessary to ensure endogenous insulin secretion was strictly suppressed after dosing. Endogenous insulin and C-peptides are released from islet β-cells; therefore, the C-peptide could be applied as a marker to measure the suppression of endogenous insulin secretion (Tao et al., 2021). In this study, C-peptide levels in all insulin formulations were inhibited, indicating the clamp test was of good quality and bioequivalence evaluation could exclude interference from endogenous insulin.

The physiological state and metabolic function of subjects of different ages may cause differences in the PK and PD indexes of insulin biosimilars (Mangoni and Jackson, 2004). The effects of the drugs in men and women were different; insulin sensitivity may vary in women during their menstrual cycles, but it is unclear whether this will affect the results of the equivalence evaluation (EMA, 2013), so we included male subjects in this study. In addition, the subject’s BMI also had an effect on subcutaneous administration, as its subcutaneous fat thickness affects drug absorption (de Galan et al., 2013). Vital signs of subjects such as blood pressure, heart rate, and body temperature can affect the absorption and metabolism of insulin biosimilars in the body by affecting blood flow at the injection site (Cengiz et al., 2014; Gradel et al., 2018). After exogenous insulin injection, exogenous insulin entered into the blood circulation and was distributed to muscles, adipose tissues, the liver, kidneys, and other organs throughout the body; ultimately about 30%–80% of exogenous insulin was degraded in the kidney; hence, the functional state of the liver and kidneys would affect the metabolism and excretion of insulin biosimilars (Iglesias and Díez, 2008). Previous studies have found that blood glucose levels affect insulin absorption because of the blood glucose–insulin feedback mechanism (Fernqvist-Forbes et al., 1988). All of these factors may have influences on insulin biosimilars and reference drugs *in vivo*, thereby affecting the bioequivalence of the two formulations. Based on the results of our study, there were differences in albumin, creatinine, T_max_, bioactive substance content, and adverse events between the equivalent and non-equivalent groups.

The results of the structural equation model revealed that adverse events and bioactive substance content significantly influence the bioequivalence. Insulin preparations were biological products; drug safety should not be ignored, especially for immune responses such as local or systemic allergic reactions. Adverse events also included abnormalities in liver and kidney function indicators, such as elevated aminotransferases or creatinine, which may affect drugs’ metabolic processes. Pharmaceutical factors were undisputed affect drug dissolution or release, including excipients or inactive ingredients that may affect drug stability, absorption, and metabolism, thereby affecting bioequivalence (Amidon et al., 1995; Rasmussen et al., 2014; FDA, 2015). In addition, pharmaceutical preparation factors can also affect the occurrence of adverse events because of their active ingredients, impurity profiles and excipients*.* Based on our findings, targeted advice was proposed as follows; first, PK and PD bioequivalence evaluation indicators should take specific drug characteristics into consideration. Second, we recommended that adverse events and active substance content should be optimized in consistency evaluation of quality and efficacy of insulin biosimilars. In preclinical studies such as animal experiments, adverse events should be strictly monitored. For products with high incidence of adverse events, a higher-level production technology should be considered to improve the purity of drugs. Differences of bioactive substance content in the preparation should be controlled within a smaller range and needed to define in further research. Third, in the process of development and production of insulin and its analog, it is necessary to strictly control the quality of biosimilars, strengthen drug production supervision, and ensure that test preparation maintains similarity with reference preparation in terms of quality, safety, and efficacy. Lastly, the subjects enrolled in the bioequivalence trials should be homogeneous. Although differences existed in albumin, creatinine, and T_max_ between the two groups, the results of the structural equation model showed that these factors had no effect on bioequivalence results. The inclusion criteria of insulin bioequivalence trials should emphasize on healthy male volunteers of normal weight aged 18–45 years; those with a BMI of 19–24 kg/m2; those without diabetes, insulin resistance and family history of diabetes; those without cardiovascular disease; and those who had no abnormalities in blood routine examinations, hepatic, and renal function tests.

Bioequivalence evaluation is a comprehensive discipline that combines the mechanism of action of drugs, the characteristics of preparations, physiological processes, and statistical methods on the basis of following laws and regulations and guiding principles. With the increasing complexity of generic drugs, technical reviews are also facing more challenges, and new evaluation methods and requirements need to be continuously studied. Since China’s accession to the ICH in 2017, China has gradually synchronized with international high-tech standards and requirements and participated in the formulation of international rules (Tang et al., 2021). Similarly, they put forward higher requirements for China’s technical evaluation. Only by continuously optimizing and improving the existing guiding principle system, we can license high-quality generic drugs faster and better.

## 5 Conclusion

In this study, we explored the factors affecting the bioequivalence of two preparations using a multiple statistical model and provided a scientific basis for the review and approval of insulin biosimilars. In addition, we proposed that adverse events and bioactive substance content should be optimized for consistency evaluation of the quality and efficacy of insulin biosimilars. Furthermore, inclusion and exclusion criteria need to be strictly executed to insure the consistency of subjects and avoid confounding factors affecting insulin biosimilar bioequivalence evaluation.

## 6 Limitations

The limitation of this study is that the sample size was not large enough, which may limit our analysis. In addition, the indicators collected in this study were inadequate, which may not comprehensively evaluate the influencing factors of bioequivalence. Female subjects were not included and unable to analyze the effect of gender. Further research should be carried out with larger samples and more factors.

## Data Availability

The datasets presented in this article are not readily available because the data from these clinical trials have not yet reached the publication stage. Requests to access the datasets should be directed to corresponding author.
